# Polyvinyl alcohol:starch:carboxymethyl cellulose containing sodium montmorillonite clay blends; mechanical properties and biodegradation behavior

**DOI:** 10.1186/2193-1801-2-376

**Published:** 2013-08-10

**Authors:** Mohammad Taghi Taghizadeh, Narges Sabouri, Babak Ghanbarzadeh

**Affiliations:** Department of Physical Chemistry, Faculty of Chemistry, University of Tabriz, P.O. Box 51666–16471 Tabriz, Iran; Department of Food Science and Technology, Faculty of Agriculture, University of Tabriz, Tabriz, Iran

**Keywords:** <Alpha>−amylase, Physical properties, Polyvinyl alcohol, Montmorillonite, Nanocomposite

## Abstract

The focuses of this study were to investigate the effect of sodium montmorillonite clay (MMT-Na) content on the physical properties and extent of enzymatic hydrolysis Polyvinyl Alcohol (PVA): Starch (S): Carboxymethyl Cellulose (CMC) nanocomposites using enzyme <alpha>−amylase. The results of this work have revealed that films with MMT-Na content at 5 wt% exhibited a significantly reduced rate and extent of starch hydrolysis. The results suggest that this may have been attributed to interactions between PVA:S:CMC and MMT-Na that further prevented enzymatic attack on the remaining starch phases within the blend. The total solids that remained after 4320 min were 65.46 wt% (PVA:S:CMC); 67.91 wt% (PVA:S:CMC:1% MMT-Na); 78.43 wt% (PVA:S:CMC:3% MMT-Na); 80.24 wt% (PVA:S:CMC:5% MMT-Na). The rate of glucose production from each nanocomposite substrates were decresed significantly as the MMT-Na percentage increased from 0 to 5% (W/W). At the level of 5% (W/W) MMT-Na, the films showed the lowest rate of glucose production values (18.95 μg/ml h). With the increase of the MMT concentration from 0 to 5%, the UTS increased 5 from 18.36 to 20.38 MPa, however, the strain to break (SB) decreased noticeably from 35.56 to 5.22%.

## Introduction

The severe environmental problems, including the increasing difficulties of waste disposal and the deepening threat of global warming (due to carbon dioxide release during incineration) caused by the nonbiodegradablility of a number of polymers (used in packaging and agriculture field) have raised concerns all over the world. To solve the problems caused by plastic waste, many efforts have been done to obtain an environmental friendly material. Most of the researches are focused on substitution of the petro-based plastics by biodegradable materials with similar properties and low in cost (Mali et al. [2005]; Mariniello et al. [2007]; Larotonda et al. [2005]). These materials not only provide the convenience for daily life but also minimize the impact to the environment after being used. In the long run, these materials will decompose into small environmentally friendly molecules and be handled in properly controlled environment (Taghizadeh et al. [2008]; Rath and Singh [1998]). In the quest for improved performance from polymers that offer biodegradation and therefore environmental acceptability, one approach is adding MMT-Na to produce nanocomposites (Averous [2004]). Blends of starch with synthetic polymers (e.g. polyvinyl alcohol, aliphatic polyesters, etc.) are prepared to achieve the desired performance for different applications. In such blends, the starch particles act as a promoter for plastic matrix biodegradation in applications such as drug delivery systems, hydrogels, bone cements and bone replacement or fixation devices (Hayashi [1994]; Pereira et al. [1998]; Teramoto et al. [2005]; Sandhu et al. [2005]; Taghizadeh and Mehrdad [2003]). PVA is a versatile polymer with many industrial applications, and it may be the only synthesized polymer whose backbone is mainly composed of C-C bonds that is biodegradable. PVA is the most readily biodegradable of vinyl polymers. It is readily degraded in wastewater activated sludge. The excellent chemical resistance, optical and physical properties of PVA resins, has resulted in its broad industrial uses (Suzuki [1979]; Watanabe et al. [1975]; Morita and Watanabe [1977]). Starch is a semicrystalline polymer stored in granules as a reserve in most plants. It is composed of repeating 1,4-α-D glucopyranosyl units: amylose and amylopectin. The amylose is almost linear, in which the repeating units are linked by α (1–4) linkages; the amylopectin has and α (1–4)-linked backbone and ca. 5% of α (1–6)-linked branches. The relative amounts of amylose and amylopectin depend upon the plant source. Corn starch granules typically contain around 70% amylopectin and 30% amylase (Pereira et al. [1998]; Teramoto et al. [2005]; Park et al. [2007]). Carboxy methyl cellulose is cellulose ether which displays thermal gelation and forms excellent films. Because of its polymeric structure and high molecular weight, it can be used as filler in biocomposite film production. Carboxy methyl cellulose can improve the mechanical and barrier properties of starch-based films (Ma et al. [2008]). In addition, it is possible to improve the mechanical properties of this polymer by adding fillers. These composites could be used in packaging where good barrier and thermal properties are required (Taghizedeh et al. [2011]). Development of the polymer/ MMT-Na nanocomposites is one of the latest revolutionary steps of the polymer technology. Preparations of blends or nanocomposites using inorganic or natural fibers are among the routes to improve some of the properties of biodegradable polymers. Further investigations on TPS:PVOH blends are of particular interest due to their excellent compatibility and improved properties such as tensile strength, elongation, toughness and processability, mainly due to an improvement in melt strength, compared to pure TPS materials (Taghizedeh et al. [2011]). Additionally, plasticisers such as maltitol, glycerol, and sorbitol, can be added to starch formulations to overcome the brittleness of these materials. Plasticizers reduced intermolecular forces and increase the mobility of polymer chains, decreasing the glass transition temperature and increasing permeability (Averous [2004]). The nanocomposites obtained from adding a low percentage of MMT-Na to polymers showed improvement in the properties such as barrier, thermal and oxidative when compared with traditional composites (Ray and Okamoto [2003]; Tjong [2006]). <alpha>−amylase are endoamylases catalyzing the hydrolysis of internal α-1, 4-glycosidic linkages in the starch in a random manner. The microbial <alpha>−amylase for industrial purposes are derived mainly from Bacillus licheniformis, Bacillus amyloliquefaciens and Aspergillus oryzae (Xiao and Yang [2006]). Bastioli et al. [1992] reported that an amylose–PVA composite (PVA-starch blend) was very slowly biodegraded and that 75% weight loss required 300 days in a degradation test with activated sludge. The biodegradable properties of these two polymers in common make them excellent pair for blending, and the water solubility of PVA makes it easy to mix evenly with the starch. All these lead to the extensive attention of the researches of PVA:starch. Enzymatic degradation tests on Starch:PVA:MMT and on its blends copolymers and various composites, have been reported (Bajpai and Shrivastava [2005]; Abbasi [2012]) however, no studies on physical properties and the enzymatic degradation of nanocomposites PVA:S:CMC and Montmorillonite were ever performed. The current paper studies the effect of sodium montmorillonite clay (MMT-Na) content on the physical properties and extent of enzymatic hydrolysis Polyvinyl Alcohol (PVA): Starch (S): Carboxymethyl Cellulose (CMC) nanocomposites using enzyme <alpha>−amylase. Mechanical properties of the blends were determined by tensile test and the modifications induced by the enzymatic treatment were evidenced by determination of weight loss, water absorption capacity, sugars released during biodegradation, as well as by UV spectroscopy and Total sugars were estimated by dinitrosalicylic acid (DNS) method (Miller [1959]).

## Materials and methods

### Materials

Starch (S) was provided by Merck company, and polyvinyl alcohol (PVA) with M_n_ = 72,000 and glycerol (Mn = 92/10, 78% purity) purchased from Merck company. Carboxy methyl cellulose sodium salt, with an average molecular weight of M_n_= 295225 was purchased from Fluka company. Sodium montmorillonite (Cloisite Na^+^) with a cation exchange capacity (CEC) of 92.6 mequiv. /100 g clay was supplied by Nanocor Inc. (Arlington Heights, IL). <alpha>−amylase (source from Bacillus subtilis) provided by sigma company. Reagent DNS was used for determination sugars released during degradation.

### Methods

The present work analyses the enzymatic degradation behavior of some montmorillonite containing nanocomposites of PVA:S:CMC based on the determinations of weight loss and the reducing sugars. The nanocomposites have been prepared from 50 wt% PVA–30 wt% S-20 wt% CMC containing small amounts of plasticizers, stabilizers and destructuring agents (stabilizers or destructuring agents such as sodium montmorillonite clay and plasticizer such as glycerol). The biodegradation studies were carried out at 37±1°C, pH = 6.8, using <alpha>−amylase for 72 h.

### Enzymatic degradation test

The enzymatic reaction mixture, comprising 1 ml of <alpha>− amylase and 25 ml of 0.1 M phosphate buffer, was placed in the clean conical flasks. The dried samples were cut into 4 × 4 cm square specimens, weighted, and immersed in the conical flasks.

The flasks were placed in a shaking incubator with a rate of 70 rpm for 72 h at 37 ± 1°C. After 1, 2, 5, 7, 9, 12, 18, 24, 30, 36, 40, 48, 54, 60 and 72 h, the samples were removed and rinsed with distilled water to remove the enzyme, dried and weighed, respectively.

The degree of enzymatic degradation (DED) was calculated as:1

where m_0_ represents the initial weight of a specimen and m_1_ is the weight of a specimen after degradation.

### Water absorption test

Pre-dried samples (with Freeze dryer FD-10 (Pishtaz Engineering Co, Iran)) were weighed for the dry weight, and then placed in a bath in distilled water at room temperature. After 1, 2, 5, 8, 24, 30 and 50 h, the samples were removed from distilled water and weighed. The water absorption capability (WAC) was calculated with the equation below:2

where m_wet_ represents the weight of the wet specimen and m_dry_ represents the weight of the dry specimen.

### Detection of reducing sugars

The Nelson–Somogyi method is one of the classical and widely used methods for the quantitative determination of reducing sugars. For sugar estimation an alternative to Nelson–Somogyi method is the dinitrosalicylic acid method-simple, sensitive and adoptable during handling of a large number of samples at a time. The reducing sugars in the degradation solutions were quantified by the dinitrosalicylic acid method: 1 ml of reagent DNS was added to 1 ml of the sample to be analysed using 1 mg/ml glucose stock solution as a standard. At the same time, the blank was prepared using 1 ml of control sample (Miller [1959]). The mixture was heated at 90–100°C for 10 min. After cooling to room temperature, 5 ml of distilled water was added, and the absorbance at 540 nm was measured. The respective carbohydrate concentration was obtained by comparison with a standard curve. Concentration of glucose produced for nanocomposites in 72 h and the first 8 h of enzymatic degradation due to acting <alpha>−amylase at temperature 37 ± 1°C.

### Mechanical properties

Ultimate tensile strength (UTS) and strain to break (SB) the films was determined at 21°C±1°C using a tensile tester (Zwick/Roell model FR010 Germany) according to ASTM standard method D882-91 (ASTM [1996]). Three dumbbelly forms films (8 cm × 0.5 cm) were cut from each of samples and were mounted between the grips of the machine. The first grip separation and cross-head speed were set to 50 mm and 5 mm/min, respectively.

### Scanning electronic microscopy (SEM)

The morphology of the surface of the films, before and after biodegradation, was investigated using a scanning electronic microscope of XL30 type (Netherland). The films were covered with pure metallic Ag. The laying down of Ag was carried out using evaporation of the metal under a high vacuum, to give a thickness of around 100 A˚.

## Results and discussion

The present study shows the role of <alpha>−amylase in PVA:S:CMC : nano-MMT degradation. Bajpai and Shrivastava [2005], who studied the biodegradation of carboxymethyl-cellulose/starch blends, found that, at small amounts of starch in the blend, a high percent of weight loss occurred while, at high starch contents, the weight loss was lower. This variation was explained in the first case, by the increase of the number of starch molecules contacting the <alpha>−amylase, so that the amount of degraded starch was higher. At high starch contents, the material becomes much more compact, which hinders the <alpha>−amylase diffusion in the polymer film.

### Weight loss and water uptake

**Figure 1:**
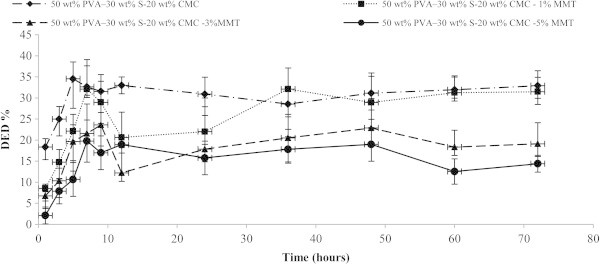
**Degree of enzymatic degradation (DED) for nanocomposite films.** Values are mean± standard deviation (*n*=3).

**Figure 2:**
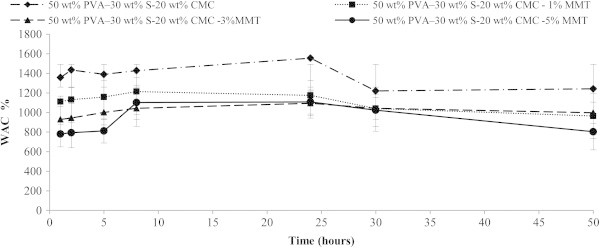
**Water absorption capability (WAC) for nanocomposite films.** Values are mean± standard deviation (*n*=3).

The water absorption capacity and the degradability are the most important properties for biodegradable materials (Arajuo et al. [2004]). The water absorption capacities of the PVA:S:CMC blend film were found to have significant difference. This was consistent with the results of Taghizedeh et al. [2011] and Abbasi [2012]. The increase of nanoparticle leads to the decrease of both weight loss and water uptake. Figures 1 and 2 clearly show that degradation is much more pronounced when the WAC% is high. In all concentrations, the addition of MMT decreased the water solubility of starch films. A comparison between the variation of the DED% and WAC% with respect to MMT-Na clay content clearly show that degradation is much more pronounced when the water sorption is high. The total solids that remained after 4320 min were 65.46 wt% (PVA:S:CMC); 67.91 wt% (PVA:S:CMC:1% MMT); 78.43 wt% (PVA:S:CMC:3% MMT); 80.24 wt% (PVA:S:CMC:5% MMT). The hydroxyl groups of MMT can form strong hydrogen bonds with the hydroxyl groups on PVA, starch and the hydroxyl and carboxyl groups on CMC; thus, improving the interactions between the molecules, the cohesiveness of biopolymer matrix and decreasing the water sensitivity. PVA:S:CMC exhibited both a high water sorption and the most significant weight loss.

### Rate and extent of glucose production

**Figure 3:**
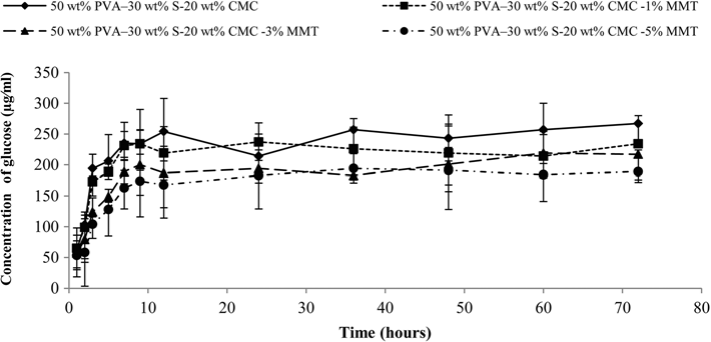
**Concentration of glucose produced for nanocomposite films in the 72 h of enzymatic degradation due to the action of <alpha>−amylase.** Values are mean± standard deviation (*n*=3).

**Figure 4:**
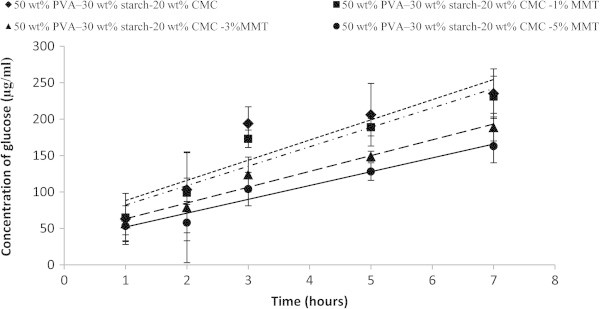
**Concentration of glucose produced for nanocomposite films in the first 8 h of enzymatic degradation due to the action of <alpha>−amylase.** Values are mean± standard deviation (*n*=3).

The rate and extent hydrolysis by acting <alpha>− amylase was measured using the DNS method glucose assay of four blends of varying MMT. Producing glucose was used as a measure of starch hydrolysis. Figure 3 shows the extent of glucose over a 72 h hydrolysis time for each substrate. Figure 4 illustrates the initial rate of glucose production by each substrate up to a hydrolysis time 8 h. The rate of glucose production was calculated; refer to Table 1 by assuming a linear relationship between glucose concentrate and time for the first 8 h of hydrolysis. The rates of glucose production from each composite substrates, were most rapid for the substrate without MMT-Na and decreased with adding MMT-Na, for PVA:S:CMC blend (27.69 μg/ml h), 26.67 μg/ml h (PVA:S:CMC:1% MMT), 21.64 μg/ml h (PVA:S:CMC:3% MMT) and 18.95 μg/ml h for (PVA:S:CMC:5% MMT). The rate of starch hydrolysis was most rapid for the substrate PVA:S:CMC and decreased with the addition of sodium montmorillonite clay. The reducing sugars in the degradation solutions, reduced by dinitrosalicylic acid, increased since the beginning until the end of the assay the relative amount of reducing sugars in the degradation solutions in similar assays without enzymes was about 100 times lower. One of the routes of biodegradation is by hydrolysis, and the enzymatic hydrolysis of starch is accompanied by the release of glucose. Figure 3 shows the release of glucose (μg/ml) during exposure to <alpha>−amylase. The free glucose increased with time for the blends showed a peak release of glucose at 8 h, followed by a decline. Obviously, the MMT has a stabilizing effect against the enzymatic attack, even after increasing the content of insoluble fraction. Each point is the mean of 3 repeats (n=3); error bars were drawn according to a 95% confidence intervals of each mean.

**Table 1:** **A summary of the rates of glucose production due to the action 1 mg of <alpha>−amylase from each substrates**

Substrate	Rate (μg⁄ml h)	R^2^
PVA:S:CMC	27.69	0.825
PVA:S:CMC:1% MMT	26.67	0.895
PVA:S:CMC:3% MMT	21.64	0.964
PVA:S:CMC:5% MMT	18.95	0.956

### Mechanical properties

**Figure 5:**
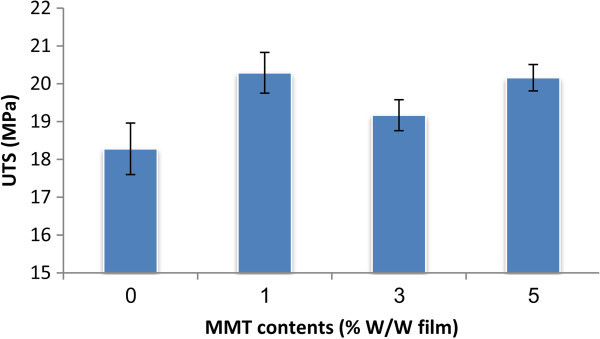
**The ultimate tensile strength (UTS) of the PVA:S:CMC:MMT films as a function of MMT content.**

**Figure 6:**
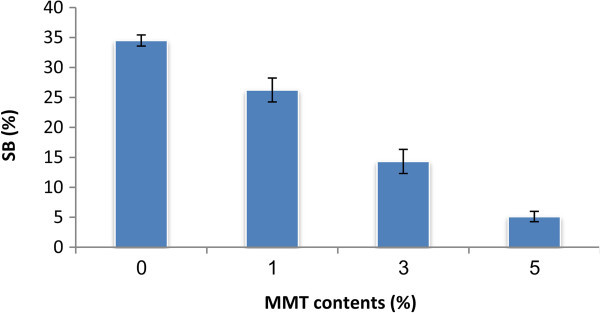
**The strain to break (SB) of the PVA:S:CMC:MMT films as a function of MMT content.**

The UTS and SB as the function of MMT concentration are shown in Figures 5, 6 and Table 2. An increase in the UTS was observed when 1- 5% (w/w) of MMT was added to the PVA/S/CMC. With the increase of the MMT concentration from 0 t o 5%, the UTS increased from 18.36 to 20.38 MPa, however, the SB decreased noticeably from 35.56 to 5.22%. A similar behavior was also observed in the UTS increment by other authors (Huang et al. [2006]; Almasi et al. [2010]; Avella et al. [2005]) in plasticized starch/ clay and starch/CMC/clay systems. This behavior was expected and was attributed to the resistance exerted by the clay itself and to the orientation and aspect ratio of the intercalated silicate layers. In addition, the stretching resistance of the oriented backbone of the polymer chain in the gallery bonded by hydrogen interaction also contributed to enhance the tensile strength. The layered silicate acts as a mechanical reinforcement of starch reducing the flexibility of the polymer. The main reason for this improvement in the mechanical properties is the stronger interfacial interaction between the matrix and layered silicate due to the vast surface exposed of the clay layers. During processing and drying of the composites, the original hydrogen bonds formed between the starch molecules were replaced by the new hydrogen bonds formed between the hydroxyl groups in PVA and starch molecules, the hydroxyl and carboxyl groups in CMC and the hydroxyl groups in MMT. The existence of these new hydrogen bonds would improve the mechanical properties.

**Table 2:** **The ultimate tensile strength (UTS) and strain to break (SB) of PVA:S:CMC:MMT films as a function of MMT content**

PVA%:starch%:CMC%	MMT (%w/w film)	UTS (MPa)	SB (%)
50%:30%:20%	0%	18.28±0.68	34.50±0.93
50%:30%:20%	1%	20.29±0.54	26.25±1.99
50%:30%:20%	3%	19.17±0.41	14.32±2.01
50%:30%:20%	5%	20.16±0.35	5.12±0.86

### Scanning electronic microscopy (SEM)

**Figure 7:**
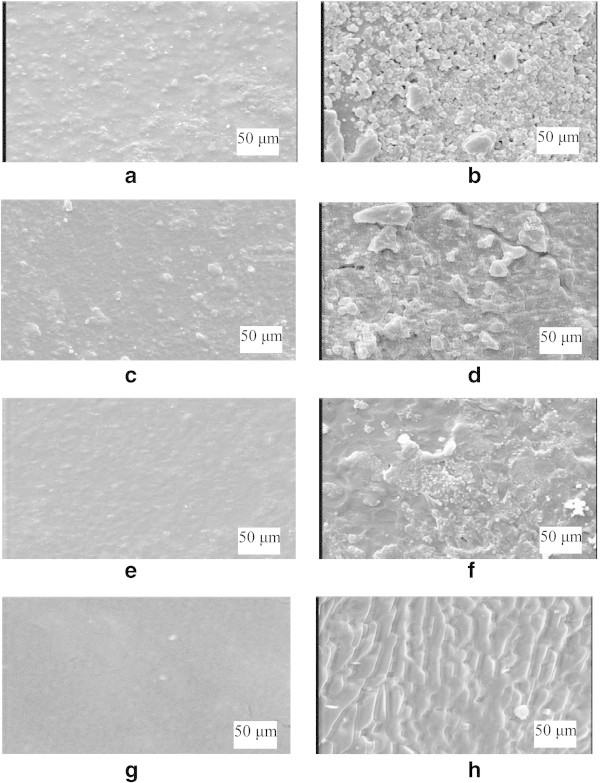
**Scanning electron micrographs of PVA:S:CMC degradable films in 72 h of enzymatic degradation due to the action of <alpha>−amylase: (a) PVA:S:CMC undegraded; (b) PVA:S:CMC degraded; (c) PVA:S:CMC with 1 wt% MMT undegraded; (d) PVA:S:CMC with 1 wt% MMT degraded; (e) PVA:S:CMC with 3 wt% MMT undegraded; (f) PVA:S:CMC with 3 wt% MMT degraded;(g) PVA:S:CMC with 5 wt% MMT undegraded; (h) PVA:S:CMC with 5 wt% MMT degraded.**

Several scanning electronic microscopy images of nanocomposites are given in Figure 7. To compare changes on surface morphology before and after degradation, Figure 7 shows the differences between the film, before degradation and after degradation. Porosity, roughness and heterogeneity surfaces decreased as a function of content MMT-Na, as detected on surface microstructure between control (smooth) and more degraded samples (72 hours) being more rough. One may observe the films are considerably destroyed, although during degradation a much more stable fibrillar fraction is revealed.

## Conclusions

The present work shows the role of MMT in physical properties and biodegradation of bionanocomposites. An increase in the Ultimate tensile strength (UTS) was observed when 1- 5% (w/w) of MMT was added to the PVA/S/CMC. With the increase of the MMT concentration from 0 t o 5%, the UTS increased 5 from 18.36 to 20.38 MPa, however, the strain to break (SB) decreased noticeably from 35.56 to 5.22%. The MMT content significantly impacted on the rate of starch solubilistion. The decrease of the degradation rate observed in the final stage can be explained to the lower degradability of the MMT-PVA-CMC domains that remain in the material. After 8–72 h, the variation is almost negligible, nearly zero as demonstrated before. The reduction of the degradation rate is also influenced by the water uptake ability of these polymers. The water uptake ability of blends was decreased significantly as the MMT percentage increased from 0 to 5% (W/W). At the level of 5% (W/W) MMT, the films showed the lowest WAC% values and DED% decreased from 27.69 μg/ml h for the film without MMT to 18.95 μg/ml h for that containing 5% MMT. Based on these results, the PVA:S:CMC:MMT bionanocomposite films show better physicochemical properties than PVA:S:CMC films and they can be potentially replaced of PVA:S:CMC films.
